# Assessment of trunk microtensiometer as a novel biosensor to continuously monitor plant water status in nectarine trees

**DOI:** 10.3389/fpls.2023.1123045

**Published:** 2023-02-15

**Authors:** María R. Conesa, Wenceslao Conejero, Juan Vera, Ma Carmen Ruiz-Sánchez

**Affiliations:** Irrigation Department, Centro de Edafología y Biología Aplicada del Segura (CEBAS-CSIC), Campus de Espinardo, Murcia, Spain

**Keywords:** automated irrigation, SPAC, stem water potential, trunk water potential, *Prunus persica* (L)

## Abstract

The objective of this work was to validate the trunk water potential (Ψ_trunk_), using emerged microtensiometer devices, as a potential biosensor to ascertain plant water status in field-grown nectarine trees. During the summer of 2022, trees were subjected to different irrigation protocols based on maximum allowed depletion (MAD), automatically managed by real-time soil water content values measured by capacitance probes. Three percentages of depletion of available soil water (α) were imposed: (i) α=10% (MAD=27.5%); (ii) α=50% (MAD=21.5%); and (iii) α=100%, no-irrigation until Ψ_stem_ reached -2.0 MPa. Thereafter, irrigation was recovered to the maximum water requirement of the crop. Seasonal and diurnal patterns of indicators of water status in the soil-plant-atmosphere continuum (SPAC) were characterised, including air and soil water potentials, pressure chamber-derived stem (Ψ_stem_) and leaf (Ψ_leaf_) water potentials, and leaf gas exchange, together with Ψ_trunk_. Continuous measurements of Ψ_trunk_ served as a promising indicator to determine plant water status. There was a strong linear relationship between Ψ_trunk_ *vs.* Ψ_stem_ (R^2^ = 0.86, *p*<0.001), while it was not significant between Ψ_trunk_ *vs.* Ψ_leaf_ (R^2^ = 0.37, *p*>0.05). A mean gradient of 0.3 and 1.8 MPa was observed between Ψ_trunk_ *vs.*Ψ_stem_ and Ψ_leaf_, respectively. In addition, Ψ_trunk_ was the best matched to the soil matric potential. The main finding of this work points to the potential use of trunk microtensiometer as a valuable biosensor for monitoring the water status of nectarine trees. Also, trunk water potential agreed with the automated soil-based irrigation protocols implemented.

## 1 Introduction

World production of peaches and nectarines (*Prunus persica* L. Batsch) has increased steadily over the last decade, ranging from 20.53 to 24.56 million metric tons (Mt) in 2010, and 2020, respectively. China alone accounts for over 45% of world peach and nectarine production, also leading in harvested area. Meanwhile, Spain leads the commercial production of peach and nectarine in the Mediterranean basin (followed by Italy), with an average of 11.58 Mt year^-1^ in the period 2015–2020 (FAOSTAST, 2022).

Water availability set the upper limit of yield productivity which is the main economic concern for growers worldwide (Fereres and Soriano, 2007). Irrigated crops are exposed to different environmental stresses during their growth and development, with drought being the most severe stress that negatively affects plant productivity (Katerji et al., 2008). The effects of drought are aggravated in arid and semi-arid areas, such as the Mediterranean region, due to the alarming depletion of water resources and the increasing demand for food due to population growth (Varela-Ortega et al., 2016; Fernández-García et al., 2020). In addition, the COVID-19 pandemic put a strain on food supply chains worldwide, so urgent and ambitious actions are needed to build more resilient agricultural systems to maximise irrigation water productivity (FAO, 2021).

Drip irrigation is probably the most important and widespread irrigation technique for improving water use efficiency, as it allows optimal use of both water and fertiliser, since they are applied directly to the root system through low-flow emitters (Burt and Styles, 2007). Another advance has been the incorporation of drip irrigation into precise irrigation agriculture, using irrigation scheduling techniques based on monitoring soil and plant water status (Vera et al., 2017; Vera et al., 2019).

Automated irrigation scheduling, based on soil water sensors that provide real-time information, has become a major challenge for precise sustainable irrigation (Vories and Sudduth, 2021). Soil water content (Ɵ_v_) is a state variable often proposed as a key input for irrigation management in decision support systems. Most of the available literature on fruit crops reported automatic irrigation controllers, using Ɵ_v_ values with on/off strategies based on real-time feedback protocols, which establish an upper and lower limit of each system state (Casadesús et al., 2012; Romero et al., 2012; Osroosh et al., 2016; Millán et al., 2019; Vories and Sudduth, 2021). In drip-irrigated nectarine trees, threshold Ɵ_v_ values converted to management allowed depletion (MAD) values were proposed to trigger/stop irrigation, thus allowing a more accurate soil-based irrigation scheduling (Vera et al., 2019). In this sense, Conesa et al. (2021) demonstrated that the automated MAD-based irrigation method, combined with regulated deficit irrigation criteria (Ruiz-Sánchez et al., 2010) proved to be a promising method for irrigation scheduling in Mediterranean agrosystems. In fact, precise deficit irrigation based on MAD threshold values used 40% less irrigation volume compared to irrigation based on conventional crop evapotranspiration (ETc) calculations (as the product of crop reference ET by local crop coefficients), maintaining yield and quality of nectarine fruits, and even increasing water use efficiency (Conesa et al., 2019).

Plant-based sensors for water status purposes address the concept of using plants as ‘biosensors’, where soil-water, atmospheric conditions and plant response are integrated (Jones, 2004). Midday stem water potential (Ψ_stem_) has been accepted worldwide as the most reliable indicator of plant water status (Abrisqueta et al., 2015). Conesa et al. (2019) proposed the long-established Ψ_stem_ as the best reference indicator of the discontinuous plant water status for drip-irrigated nectarine trees. However, Ψ_stem_ is a very labour-demanding and destructive measurement that cannot be automated.

Nowadays, IoT in agriculture has led to the development of many detection methods as plant indicators to measure water status and to assess plant responses to environmental stresses. Indicators of plant water status on a continuous basis include those based on sap flow and stem heat balance (Smith and Allen, 1996; Navarro et al., 2020; Dix and Aubrey, 2021), trunk diameter fluctuations (Fernández and Cuevas, 2010; Ortuño et al., 2010), and leaf turgor (Martínez-Gimeno et al., 2017; Padilla-Díaz et al., 2018). However, although the latter two are non-invasive techniques (Fernández, 2014), the equipment used requires a significant labour input to properly monitor plant water status, as well as specialised staff for data processing.

The emerging sensors identified as microtensiometers (MTs) are embedded in the tree trunk and directly measure the trunk water potential (Ψ_trunk_) on a continuous basis, which is a major advantage over discrete Ψ_stem_ determinations. This sensor is a microelectromechanical system-based microtensiometer that measures plant water status with a high degree of accuracy. It can be automated and provides easy-to-interpret continuous data, in pressure units comparable to those of the Ψ_leaf_ or Ψ_stem_ acquired with traditional pressure chamber methods (Pagay et al., 2014; Lakso et al., 2022).

To our knowledge, only a few studies have addressed the performance of these MTs sensors in field conditions and under different water availability scenarios (*e.g*. Blanco and Kalcsits (2021) in apple and Pagay (2022) in gravepines). Our hypothesis is that MTs can provide stable continuous Ψ_trunk_ data, and we seek to know if they can be used to validate automated MAD-based irrigation protocols, as we have already done from discrete Ψ_stem_ determinations in previous experiences (Conesa et al., 2019; Vera et al., 2019, Conesa et al., 2021; Mira-García et al., 2021).

This study aims to validate the use of Ψ_trunk_ as a continuous plant-based water status indicator in drip-irrigated nectarine trees grown under Mediterranean conditions threatened by water scarcity. Irrigation scheduling was automatically managed by real-time Ɵ_v_ values at different levels of MAD corresponding to well-irrigated, moderate deficit and drought conditions. The performance of MAD-based irrigation method was also analysed in the soil-plant-atmosphere continuum

## 2 Material and methods

### 2.1 Field conditions

The experiment was carried out from June to September in 2022, in a 0.5 ha orchard of twelve-year-old early-maturing nectarine trees (*Prunus persica* (L.) Batsch, cv. Flariba, on GxN-15 rootstock), at the CEBAS-CSIC experimental station, Murcia (Spain, 38° 06’ 31’’ N, 1° 02’ 14’’ W). The trees were spaced 6.5 m x 3.5 m and trained to an open-centre canopy. The soil in the 0-0.5 m layer was stony and shallow with a clay-loam texture and low organic matter content 1.3%. The average bulk density was 1.43 g cm^-3^. Soil water content (Ɵ_v_) at field capacity and at permanent wilting point was 0.29 and 0.14 m^3^ m^-3^, respectively. The drip-irrigation system consisted of one dripline per row of trees with four pressure-compensated emitters (4 l h^-1^) per tree located 0.5 and 1.3 m from the tree trunk. The amount of water applied was measured with a pulse flowmeter (Sensus, 120 HRI-A, Barcelona, Spain).

Seasonal fertiliser applications were 83, 56, and 109 kg ha^−1^ of N, P_2_O_5_ and K_2_O, respectively, applied by fertigation system (Vera and de la Peña, 1994). Other usual cultural practices (e.g. weed control, fertilization, pruning, fruit thinning) were carried out following the recommendations of commercial fruit tree orchards.

The experiment consisted of an automated soil-based irrigation treatment, managed according to different irrigation criteria (see 2.4 section), which were randomly distributed in four replicates, each consisting of six nectarine trees (n= 24). Measurements of soil and plant water relations were taken on a representative tree from each replicate.

### 2.2 Agrometeorological status

During the experimental period, agrometeorological data (air temperature, T_a_; relative humidity, RH; and rainfall) were recorded every 15 min by an automatic weather station located in the CEBAS-CSIC experimental field, next to the nectarine tree orchard (http://www.cebas.csic.es/general_spain/est_meteo.html). Hourly reference crop evapotranspiration (ET_0_, mm) was calculated following the Penman-Monteith equation (Allen et al., 1998). Vapour pressure deficit (VPD, kPa) was calculated from daily maximum T_a_and minimum RH.

The hourly air water potential (Ψ_air,_ *MPa*) was calculated with the equation (Nobel, 1983):


(1) $$\Psi_{air}=\;\frac{R\;\cdot \;T}{V_{w}}\ln\frac{RH}{100}$$

where, R is the gas constant (R=0.082 atm L K^-1^ mol^-1^), T is the absolute temperature (273+T_a_, °C), V_w_ the partial molar volume of water in the atmosphere (18 cm^3^ at 20 °C), and RH is the air relative humidity (%).

### 2.3 Soil water status

Soil water status was continuously monitored by measurements of soil water content (Ɵ_v_) and soil matric potential (Ψ_m_), as follows:

#### 2.3.1 Soil water content

Volumetric soil water content (Ɵ_v,_ %) was monitored with multi-depth EnviroScan^®^ capacitance probes (Sentek Sensor Technologies, Sidney, Australia). Four PVC access tubes were installed 10 cm from the emitter located close (0.5 m) to the tree trunk in four representative trees (one in each replicate). Each capacitance probe had sensors at 0.1, 0.3, 0.5, and 0.7 m depth, and was connected to a radio transmission unit. Values were read every 5 min and the average was recorded every 15 min. The probes were normalised and calibrated following the procedure proposed by Starr and Paltineanu (2002). Drip gauges (Pronamic, Ringkoebing, Denmark) were installed below the emitter near the capacitance probe to monitor real-time irrigation amounts and to detect any flow rate failures during the irrigation events. The radio-transmission units sent the data to a gateway that is connected to the addVANTAGE cloud server (ADCON Telemetry, Vienna, Austria) for data acquisition, processing, and visualisation.

#### 2.3.2 Soil matric potential

Soil matric potential (Ψ_m,_ kPa) was measured with digital tensiometers (WEENAT, Nantes, France) consisting of granular matrix sensors, which were installed in the wet bulb of two nectarine trees, at 0.3 and 0.6 m soil depth. Data were recorded and visualised on the cloud platform www.weenat.com.

### 2.4 MAD-based irrigation protocol

Average Ɵ_v_ values of the 0-0.5 m soil profile, representing the active water uptake of the roots (Abrisqueta et al., 2017), were used to act on electro-valves by means of the telemetry network (see 2.3.1 section). The maximum allowable depletion (MAD) values were established as irrigation threshold Ɵ_v_, as derived from the concept proposed by Merriam (1966), as:


(2) $$MAD=\;FC-\alpha \;\frac{\left(FC-WP\right)}{100}\;$$

where, FC is the field capacity, WP is the wilting point, α is the percentage depletion of available water in the soil.

In the experiment, the following α criteria were applied:

α = 10%: well-irrigated, from 3 to 29 June 2022.
α = 50%: moderate soil water deficit, from 30 June to 29 July 2022.
α = 100%: severe soil water deficit. No irrigation was applied from 30 July to 1 September 2022.

Recovery: Irrigation recovered at full crop water requirements, when Ψ_stem_ reached -2.0 MPa, from 2 September to 30 September 2022.

### 2.5 Plant water status

During the experimental period, plant water status was estimated by weekly measurements of discrete plant-based water indicators: leaf and stem water potentials and leaf gas exchange. In addition, daily time-courses were made on representative days of the well-irrigated period (23 June 2022, DOY 174), at the end of the moderate water deficit (α=50%) period (29 July 2022, DOY 210), and at the end of drought (α=100%) period (1 September 2022, DOY 244). All measurements were always performed on one leaf of the same trees in each replicate (n=4). In addition, trunk water potential was measured continuously in two of the four replicates (n=2).

#### 2.5.1 Leaf and stem water potentials

Leaf (Ψ_leaf_, MPa) and stem (Ψ_stem,_ MPa) water potentials were measured on four leaves (one leaf per replication) at midday (13:00-14:00 h, GMT+2), and hourly during daily courses on fully expanded healthy leaves, using a pressure chamber (Soil Moisture Equipment Corp. Model 3000, Santa Bárbara, CA, USA) as recommended by Turner (1988). Measurements of Ψ_leaf_ were made in sunny, freely transpiring leaves, while for Ψ_stem_, leaves were located on the shaded side of the tree, close to the tree trunk, and covered with aluminium foil for at least 2 h before the determinations (McCutchan and Shackel, 1992). Both measurements were carried out weekly during the experiment, as well as hourly in the daily time-courses.

#### 2.5.2 Trunk water potential

Trunk water potential (Ψ_trunk,_ MPa) was determined using microtensiometers (MTs; FloraPulse, Davis, CA, USA, www-florapulse.com) embedded directly into the trunk on the shaded side of two nectarine trees, at 0.4 m from soil surface (**Illustration 1A**). Installation of the MTs was carried out according to the recommendations of the manufacturer. The technical details given by Pagay et al. (2014); Black et al. (2020), and Lakso et al. (2022) were also considered. The sensors were allowed to equilibrate with the tree (through the mating compound) within 2 days of installation (Pagay, 2022). Trunk water potential (Ψ_trunk_) data were obtained every 15 min, and transmitted using the same telemetry network (ADCON Telemetry, Vienna, Austria) (**Illustration 1B**).

**Illustration 1:**
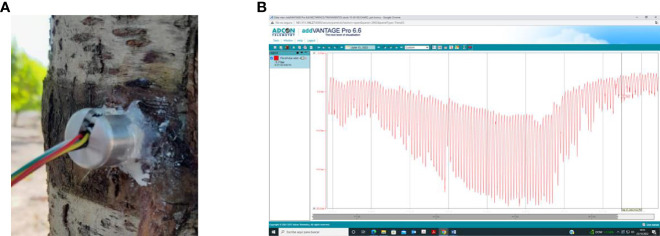
**(A)** MT sensor installed in the nectarine tree trunk, and **(B)** data visualisation of Ψ_trunk_ on addVANTAGE web server.

#### 2.5.3 Leaf osmotic potentials

Leaf osmotic potentials (Ψ_π,_ MPa) were determined at predawn, midday and afternoon on the same leaves used for Ψ_leaf_ determinations, coinciding with daily time-courses. Leaves were frozen in liquid nitrogen and the osmotic potential was measured after thawing the samples and expressing sap by using a vapour pressure osmometer (model WESCOR-5520; Wescor Inc., Logan, UT, USA) following the recommendations of Gucci et al. (1991). Leaf turgor potentials (Ψ_t,_ MPa) at predawn, midday and afternoon were calculated as the difference between osmotic and leaf water potentials. Leaf osmotic potential at full turgor (Ψ_π100,_ MPa) was measured on leaves adjacent to those used for Ψ_leaf_ at predawn. The leaves were excised and placed by their petioles in distilled water overnight to reach full saturation, after which they were frozen in liquid nitrogen (-196 °C) and stored at -30 °C, following the same methodology as for Ψ_π_. The osmotic adjustment was estimated by comparing Ψ_π100_ values at α=10% (well-irrigated), and α=100% (non-irrigated).

#### 2.5.4 Leaf gas exchange

Net photosynthesis (P_n_, μmol m^−2^ s^−1^), stomatal conductance (g_s_ mmol m^−2^ s^-1^), and transpiration rate (E, mmol m^−2^ s^−1^) were measured on one mature sunny leaf per replication (n=4) in the early morning (9:00-10:00 h, GMT+2), using a portable gas exchange system (LI-COR, LI-6400) at photon flux density (PPFD) ≈ 1500 μmol m^−2^ s^−1^ and CO_2_ concentration ≈ 400 μmol mol^−1^. During daily time-courses, hourly leaf gas exchange measurements were taken under ambient PPFD conditions at the time of measurements. Transpiration efficiency (WUE_T_, μmol mmol^-1^) was calculated as the P_n_/E ratio.

### 2.6 Sensitivity analysis

For the plant-based status indicators, the signal intensity (SI) was calculated as the ratio between all data registered at α=100% (drought conditions) and α=10% (well-irrigated conditions) periods. To determine noise, the coefficient of variation (CV) of the measurements was calculated for each indicator.

Sensitivity was determined using two algorithms:

- Traditional method (S), as proposed by Goldhamer and Fereres (2001):

(3) $$S=\frac{SI}{CV}$$

S is always greater than 0, and the higher the value, the greater the sensitivity.

- Corrected sensitivity (S*), as proposed by De la Rosa et al. (2014).

(4) $$S^{*}=\frac{\left(SI-1\right)}{CV}$$

The interpretation of the values obtained with this algorithm is as follows:

(a) S* > 1: indicates sensitivity to water deficit.
(b) 1 > S* > 0: The noise is greater than the increase in signal intensity.
(c) S* = 0: not sensitive to water deficit.
(d) S*< 0: anomalous behavior.

### 2.7 Statistical analysis

Data were depicted using the SigmaPlot v. 14.5 software (Inpixon, PA, USA). Statistical comparisons were considered significant at *p<*0.05, using Pearson’s correlation coefficient. Relationships between indicators of plant and soil water status were explored by linear regression analyses. The coefficient of determination (R^2^) and mean squared error (MSE) were used to assess the goodness of fit. All analyses were performed with SPSS v. 9.1 (IBM, Armonk, NY, USA).

## 3 Results

### 3.1 Automated control of irrigation and climatology

**Figure 1:**
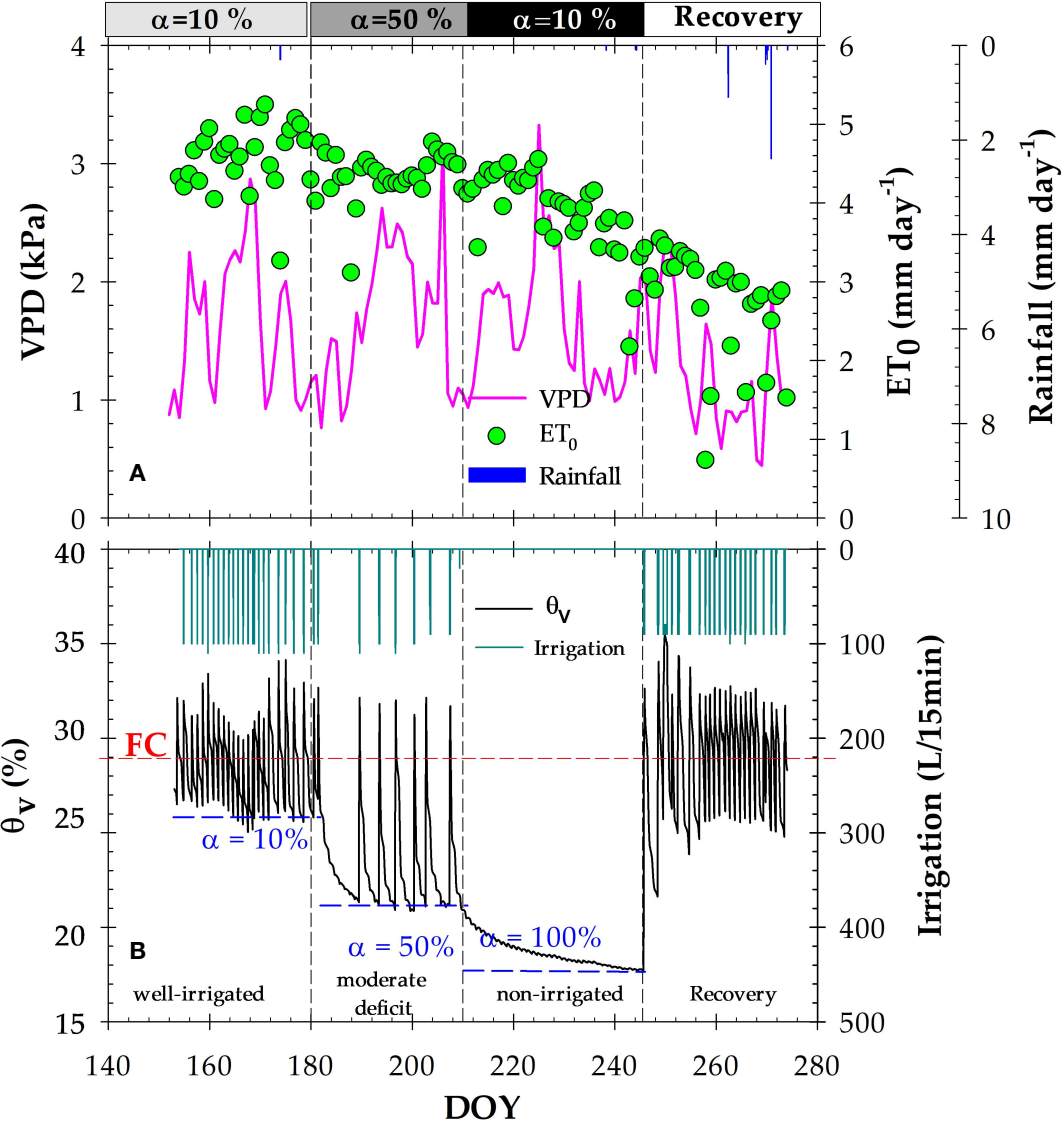
**(A)** Daily vapour pressure deficit (VPD, kPa), reference crop evapotranspiration (ET_0_, mm), and rainfall (mm); **(B)** Soil water content (θ_v_, %) in the 0–0.5 m soil profile, and irrigation events (mm), during the experimental period. The dashed horizontal red line corresponds to the field capacity (FC), and the dashed blue lines indicate the soil water deficit (α) criteria: 10% (well-irrigated), 50% (moderate deficit) and 100% (severe deficit, non-irrigated), respectively. The dashed vertical lines delimit each irrigation criterion. DOY: Day of the year.

The climatic conditions during the experiment, comprising the postharvest period of the early-maturing nectarine trees (June to October), corresponded to a typical Mediterranean semi-arid summer environment, high values of ET_0_ (472.1 mm) and low rainfall (10.2 mm concentrated during the recovery period). Daily VPD values varied in a range of 0.2 and 3.3 kPa, representing the greatest day-to-day variability of the agrometeorological variables studied (**Figure 1A**).

Volumetric soil water content (Ɵ_v_) fluctuated in response to irrigation, root water uptake and rainfall events. Furthermore, Ɵ_v_ in the active root zone (0-0.5 m depth) was clearly influenced by the different imposed MAD-based protocols (**Figure 1B**). At α=10%, MAD=27.5% (well-irrigated conditions) induced by daily irrigation frequency, Ɵ_v_ values varied around field capacity (FC), increasing slightly above this value at the end of each irrigation event. At α=50%, MAD=21.5% (moderate soil water deficit) induced an irrigation frequency of 2 or 3 day. When irrigation water was withheld (α=100%), Ɵ_v_ decreased until the minimum value of Ɵ_v_ ≈ 17%, close to the wilting point value. Subsequently, during the recovery period, Ɵ_v_ reached variable FC values in response to irrigation and, to a lesser extent, rainfall events. The total amount of irrigation applied during the experiment (including the recovery phase) was 109.5 mm (**Figure 1B**).

### 3.2 Seasonal soil-plant-atmosphere water indicators

**Figure 2:**
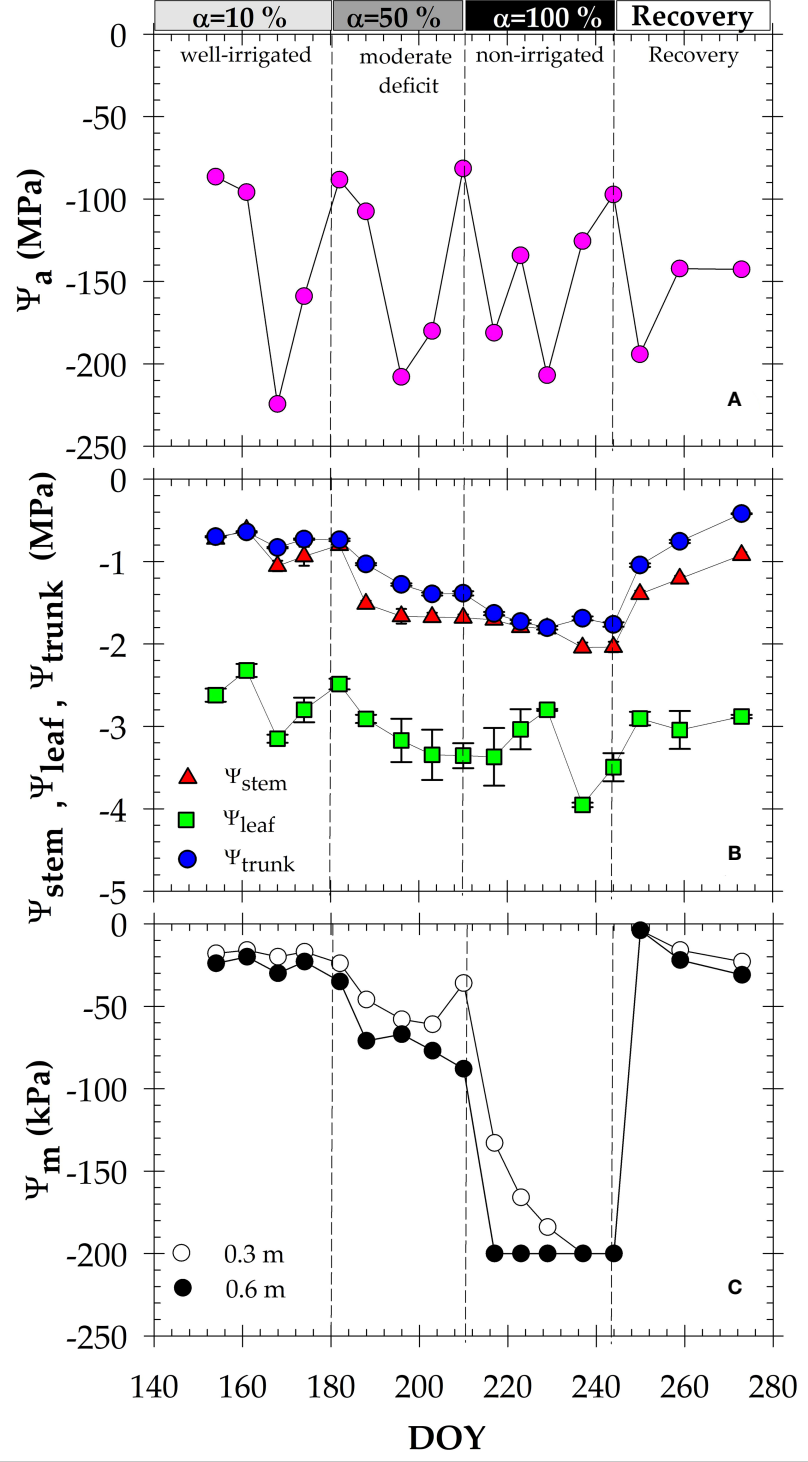
Seasonal course of: **(A)** daily mean air water potential (Ψ_air_); **(B)** midday stem (Ψ_stem_), leaf (Ψ_leaf_) and trunk (Ψ_trunk_) water potentials, and **(C)** soil matric potential (Ψ_m_) at 0.3 and 0.6 m of the soil profile. Each point is the average of four leaves, two MTs, and two granular matrix sensors. Vertical bars at data points are ± SE (not shown when smaller than the symbols). Dashed vertical lines delimit each irrigation criterion. DOY: Day of the year.

The data in **Figure 2** show the seasonal course of water status in the soil-plant-atmosphere continuum (SPAC). The seasonal trend of air water potential (Ψ_air_) was highly variable from day-to-day during the study, with a maximum value of -81.5 MPa (DOY 210, α=50%) and minimum of -224 MPa (DOY 168, α=10%) (**Figure 2A**).

Soil water potential from the granular matrix sensors (Ψ_m_), assuming osmotic and gravitational components to be negligible, ranged from -4 ± 0.85 to -26 ± 1.26 kPa at both depths explored (0.3 and 0.6 m) under well-irrigated conditions (α=10%). Under moderate deficit conditions (α=50%), Ψ_m_ decreased, showing slightly lower values at 0.6 than at 0.3 m, and reaching minimum values of -61 ± 3.45 and -77 ± 4.48 kPa (MPa) at 0.3 and 0.6 m, respectively. When irrigation was suspended (α=100%), Ψ_m_ continued to decrease, reaching its minimum allowable reading (-200 kPa) only one week later at 0.6 m depth, and after 13 days of withholding irrigation at 0.3 m (**Figure 2C**).

Plant water potentials evaluated at three canopy levels (leaf, stem and trunk) reflected the different MAD applied during the experiment (**Figures 2A, B**). Both Ψ_stem_ and Ψ_trunk_ exhibited a constant pattern during α=10%, averaging -0.83 ± 0.09 and -0.73 ± 0.06 MPa, respectively, during this well-irrigated period. In accordance with the imposed soil water deficit, the trend of both plant indicators decreased, reaching the minimum values of Ψ_stem_ = -2.04 ± 0.06 MPa and Ψ_trunk_ = -1.81 ± 0.29 MPa, at the end of α=100%. A more irregular trend was observed for Ψ_leaf_ during the experiment, showing lower values than those of Ψ_stem_ and Ψ_trunk_, and minimum values of -3.95 ± 0.26 MPa at the end of the irrigation withholding phase (DOY 237, α=100%).

**Figure 3:**
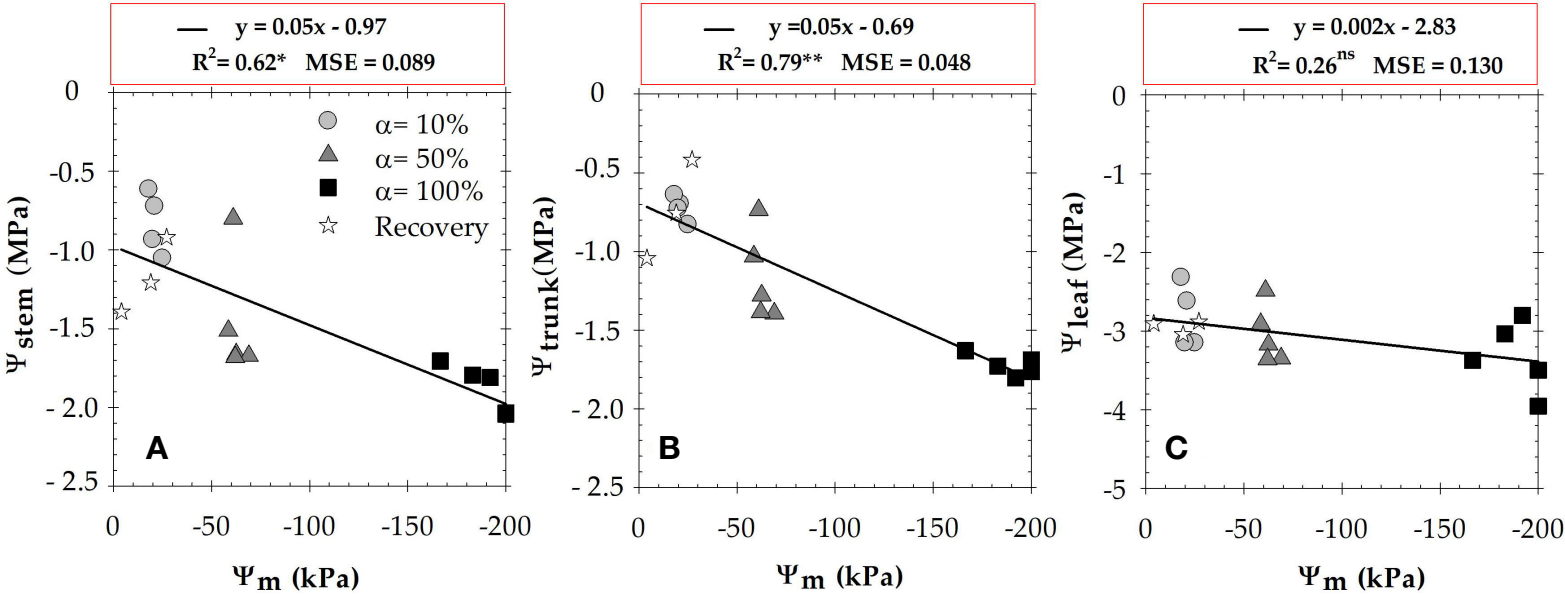
Relationship between the midday values of soil matric potential (Ψ_m_) (average of 0.3 and 0.6 m), and **(A)** stem water potential (Ψ_stem_); **(B)** trunk water potential (Ψ_trunk_); and **(C)** leaf water potential (Ψ_leaf_), during the experimental period. The different symbols correspond to the four irrigation criteria. Each point is the mean of four leaves and two matrix sensors. R^2^ is the coefficient of determination. *: *p* ≤ 0.05 **: *p* ≤ 0.01, ns: not significant. MSE: mean squared error.

Correlation analysis between soil and plant water potentials showed a close linear relationship with the highest dependence found between Ψ_m_ and Ψ_trunk_ (R^2^ = 0.79), and the lowest (not significant) between Ψ_m_ and Ψ_leaf_ (R^2^ = 0.26) (**Figure 3**). However, there was no significant correlation between Ψ_air_ and plant water potentials (data not shown).

**Figure 4:**
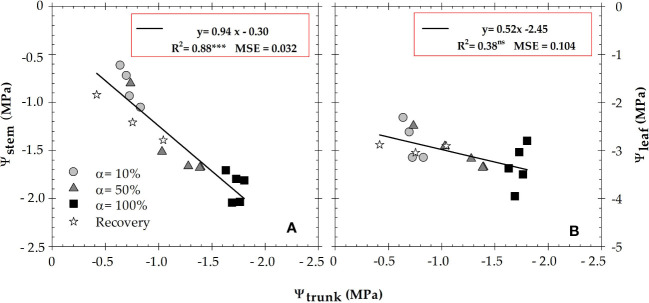
Relationship between midday values of trunk water potential (Ψ_trunk_) and **(A)** stem water potential (Ψ_stem_), and **(B)** leaf water potential (Ψ_leaf_), during the experimental period. The different symbols correspond to the different irrigation criteria. Each point is the mean of four leaves and two matrix sensors. R^2^ is the coefficient of determination. ***: *p* ≤ 0.001, ns: not significant. MSE: mean squared error.

During the experiment, the gradient between midday values of Ψ_stem_ and Ψ_trunk_ varied over a range of 0.02 to 0.5 MPa, while this gradient was higher for Ψ_leaf_ and Ψ_trunk_ (1.0 to 2.5 MPa) (**Figure 2B**). In this regard, Ψ_trunk_ data obtained with microtensiometers (MTs) were correlated with the plant-based indicators measured with a pressure chamber: Ψ_stem_ and Ψ_leaf_ (**Figure 4**). The results indicated a robust significant correlation between Ψ_trunk_ to Ψ_stem_ (R^2^ = 0.86), and, again, to a lesser extent between Ψ_trunk_ to Ψ_leaf_ (R^2^ = 0.37).

**Figure 5:**
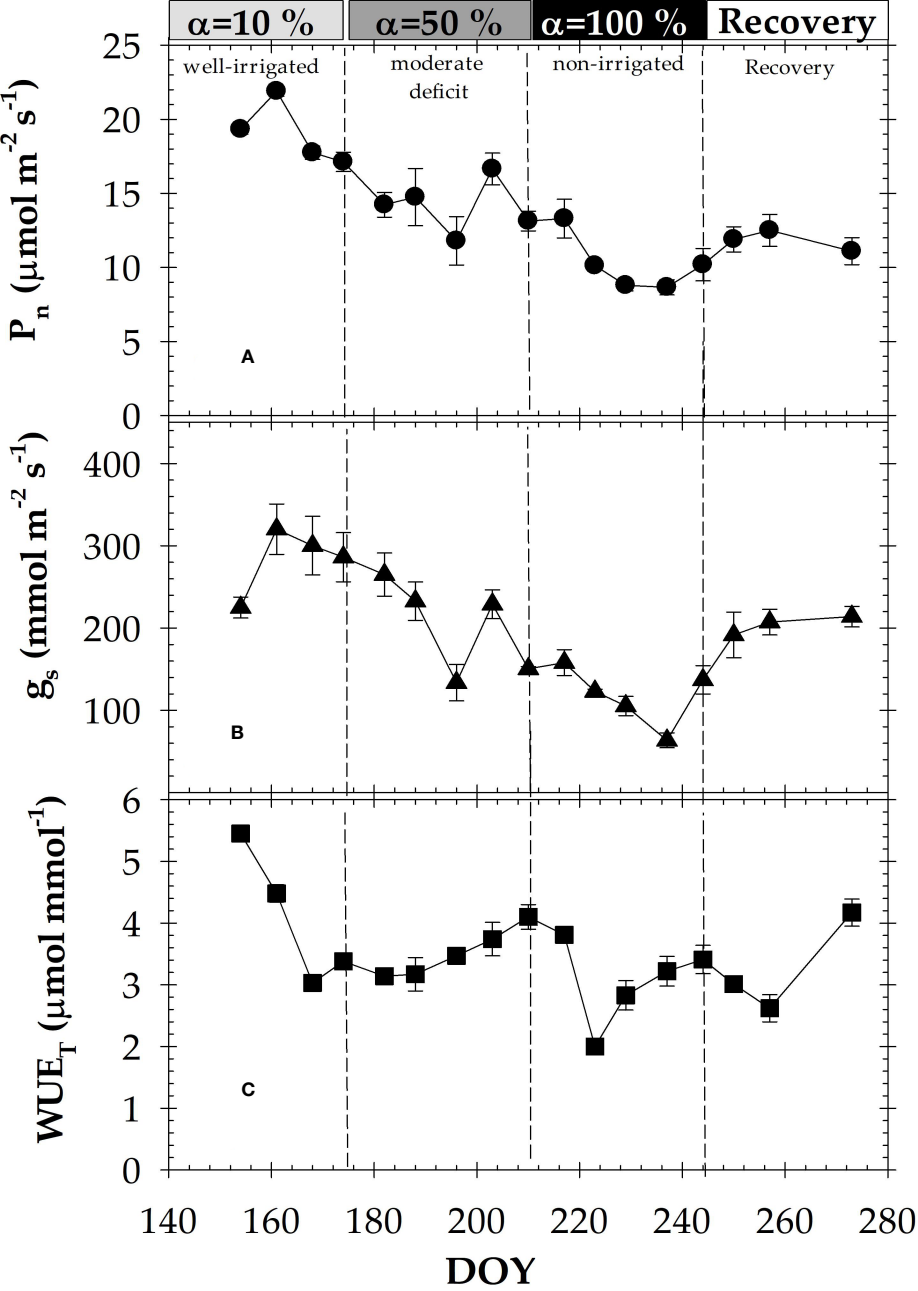
Seasonal time-course of: **(A)** net photosynthesis (P_n_); **(B)** stomatal conductance (g_s_); and **(C)** transpiration efficiency (WUE_T_) during the experimental period. Each point is the mean of four leaves. Vertical bars in data points are ± SE (not shown when smaller than the symbols). Dashed vertical lines delimit each irrigation criterion. DOY: Day of the year.

Leaf gas exchange (P_n_ and g_s_), measured simultaneously with stem and leaf water potentials, showed a seasonal trend that mirrored the soil deficit imposed by the MAD-irrigation protocols (**Figures 5A, B**). At α=10%, both P_n_ and g_s_ reached their maximum values of about 22 ± 0.34 µmol m^-2^ s^-1^ and 320 ± 30.5 mmol m^-2^ s^-1^, respectively. The lowest values of P_n_ (8.6 ± 0.51 µmol m^-2^ s^-1^) and g_s_ (63.5 ± 9.05 mmol m^-2^ s^-1^) were obtained at the end of the α=100% period (severe water deficit). P_n_ and g_s_ also varied in response to plant water potentials under quite contrasting environmental conditions (**Figure 2B**). Values of WUE_T_ increased with the imposed soil water deficit (**Figure 5C**), reaching a maximum value of 5.5 ± 0.10 µmol mmol^-1^. (**Figures 5C**). It is also important to note that despite irrigation being re-established during the recovery phase, the mean values of P_n_ and g_s_ were lower than those obtained under well-irrigated conditions (α=10%).

### 3.3 Diurnal indicators of soil-plant-atmosphere water status

**Figure 6:**
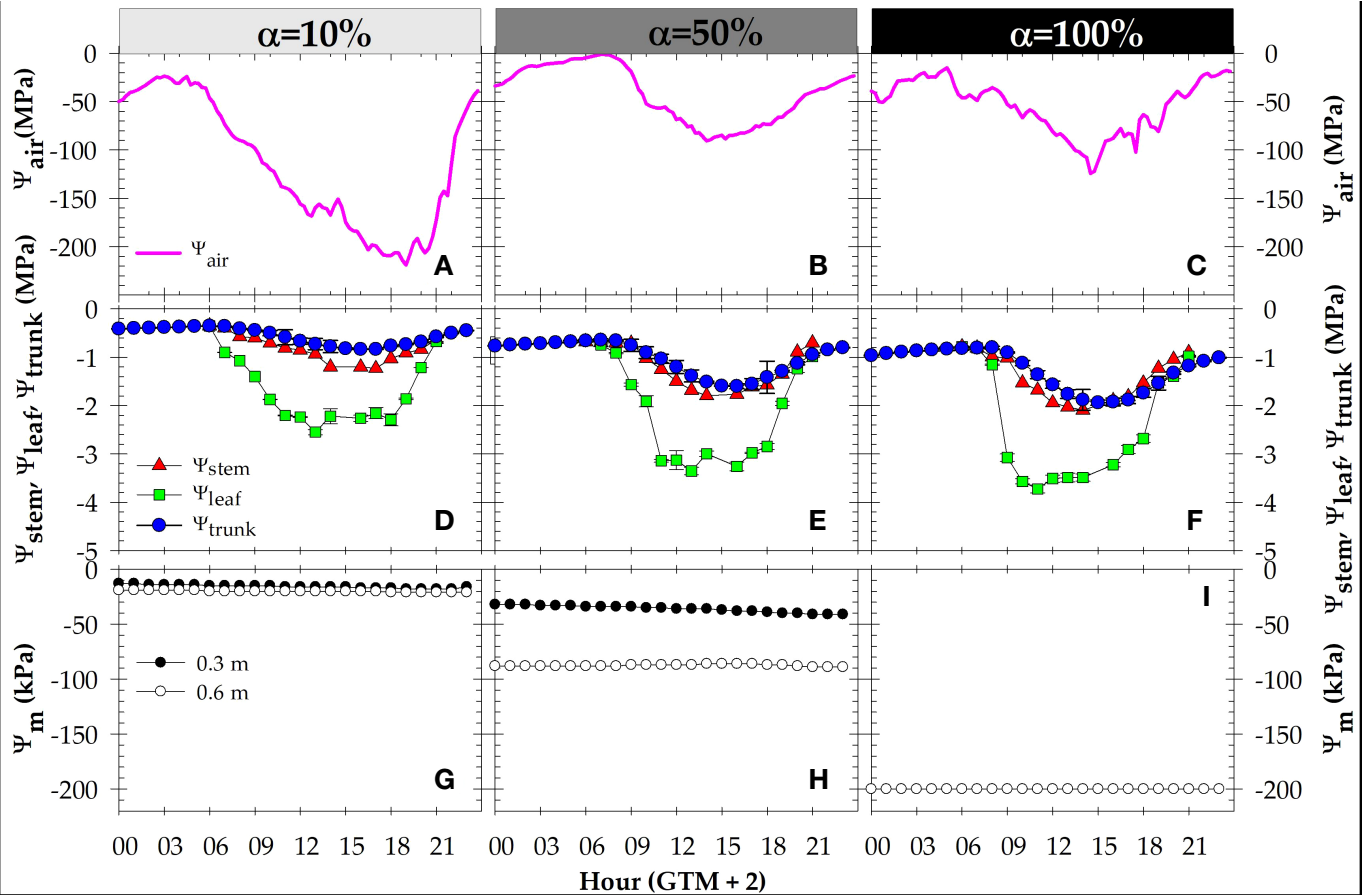
Daily time-courses of: **(A-C)** air water potential (Ψ_air_); **(D-F)** leaf (Ψ_leaf_), stem (Ψ_stem_), and trunk (Ψ_trunk_) water potentials; and **(G-I)** soil matric potential (Ψ_m_) at 0.3 and 0.6 m in the soil profile, during different irrigation criteria: α=10% (DOY 174), α=50% (DOY 210) and α=100% (DOY 244). Each point is the mean of four leaves, two MTs, and two granular matrix sensors. Vertical bars in the data points are ± SE (not shown when smaller than the symbols). GMT: Greenwich mean time.

The daily time-course of soil-plant-atmosphere water status indicators were evaluated on representative days of the well irrigated period (23 June 2022, DOY=174), at the end of moderate water deficit (α=50%) period (29 July 2022, DOY=210), and at the end of drought (α=100%) period (1 September 2022, DOY=244) covering the whole daily light period (06:00 to 21:00 h). The values of soil water content during well irrigated period were 27.82 ± 0.49; 39.54 ± 0.35; 26.63 ± 0.28 and 33.50± 0.19% at 0.1, 0.3, 0.5, and 0.7 m of soil depth, respectively. Meanwhile, at α=50%, and α=100%, θ_v_ decreased up to 35% below the FC values, mainly affecting the upper soil depth (> 0.5 m) with little variation observed at the deeper layer (data not shown). In addition, Ψ_m_ remained constant during each daily course, decreasing as water deficit increased (**Figures 6G–I**).

Agrometeorological conditions changed greatly during the days selected for punctual measurements (**Figures 6A–C**). A very demanding day coincided with the well-irrigated period (α=10%), being the warmest of the three diurnal courses studied, with minimum Ψ_air_ values of -218 MPa registered in the early afternoon. Sunny mild-demanding days corresponded to the end of α=50% and α=100% periods, when minimum Ψ_air_ values of -84 and -124 MPa were recorded at midday, respectively.

The diurnal patterns of plant water potentials mirrored the imposed soil water deficit based on MAD-threshold values (**Figures 6D–F**), despite the different climatic conditions observed. At α=10%, minimum values of -0.87 ± 0.08, -1.30 ± 0.06 and -2.1 ± 0.31 MPa were measured in the early afternoon (16:00 h GMT+2) for Ψ_trunk_, Ψ_stem_, and Ψ_leaf_, respectively. At α=50%, plant water potentials recorded their minimum values at different times of the day. In this sense, Ψ_leaf_ and Ψ_stem_ obtained their minimum values at midday: -3.3 ± 0.26 MPa (Ψ_leaf_) and -1.8 ± 0.12 MPa (Ψ_stem_), whereas the minimum value of Ψ_trunk_ (-1.6 ± 0.19 MPa) was obtained in the afternoon (17:00 GMT+2). The severe water deficit situation recorded at α=100% induced a decrease in plant water potentials from predawn onwards. In this period, Ψ_leaf_ and Ψ_stem_ reached again their minimum values at midday (-3.5 ± 0.26, and -2.1 ± 0.12 MPa, respectively); and those for Ψ_trunk_ (-1.9 ± 0.21 MPa) in the afternoon (17:00 GMT+2) (**Figures 6D–F**). It must be emphasized that the values of water potentials at predawn decreased from -0.35 ± 0.08, -0.68 ± 0.03 to -0.80 ± 0.11 MPa, at α=10, 50, and 100% periods, respectively.

**Figure 7:**
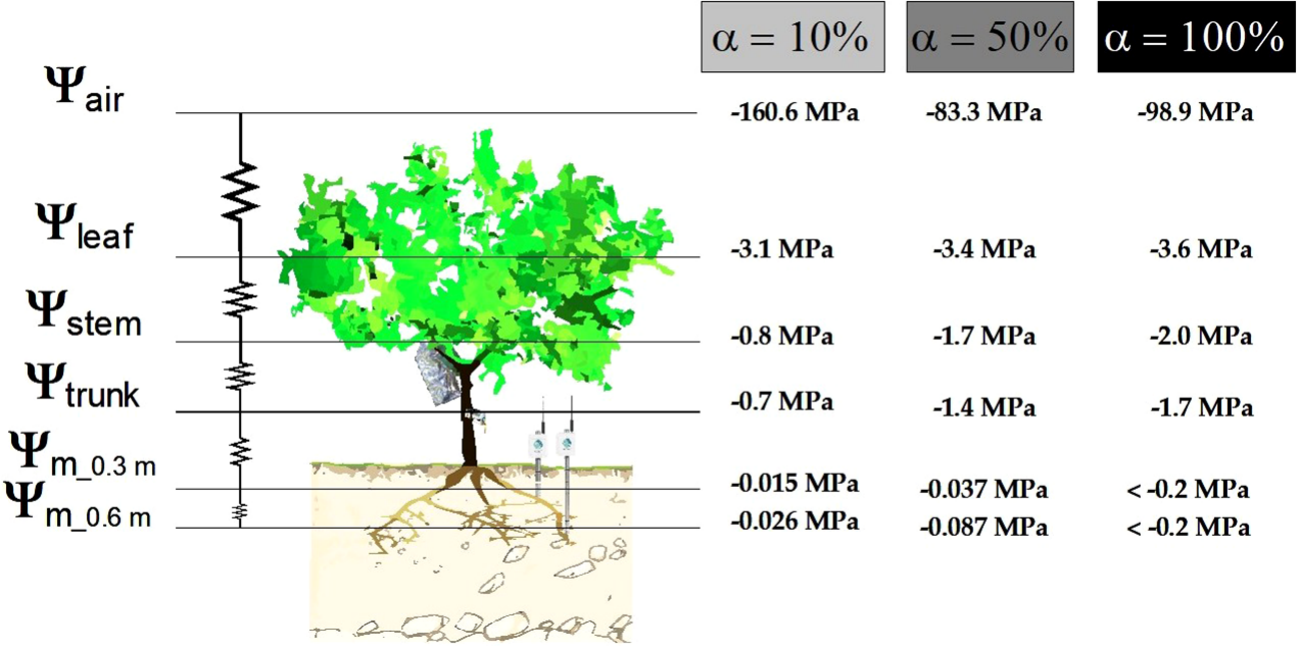
Mean values of water potential at midday in the SPAC during each irrigation criterion: α=10% (well-irrigated), α=50% (moderate water deficit), and α=100% (severe water deficit, non-irrigated).

To represent the SPAC resistances to water flow along the soil-plant-atmosphere continuum, the experimental values of water potentials at midday were drawn (**Figure 7**). It can be observed that the highest gradient was found from leaf to air, which is tuned by stomatal aperture, regulating the change of water state from liquid to gas, while the lowest gradient (0.3 MPa) was between Ψ_trunk_ and Ψ_stem_. Under well irrigated conditions, the next important gradient was between Ψ_stem_ and Ψ_leaf_ (1.7 MPa), followed by Ψ_m_ to Ψ_trunk_ gradient (0.7). As the water deficit progresses, these gradients increase, especially in the case of root to trunk water potential differences (1.5 MPa at the end of the non-irrigation period), and remained almost constant for Ψ_stem_ to Ψ_leaf_ gradient, and even decreased for leaf to air water potentials.

**Figure 8:**
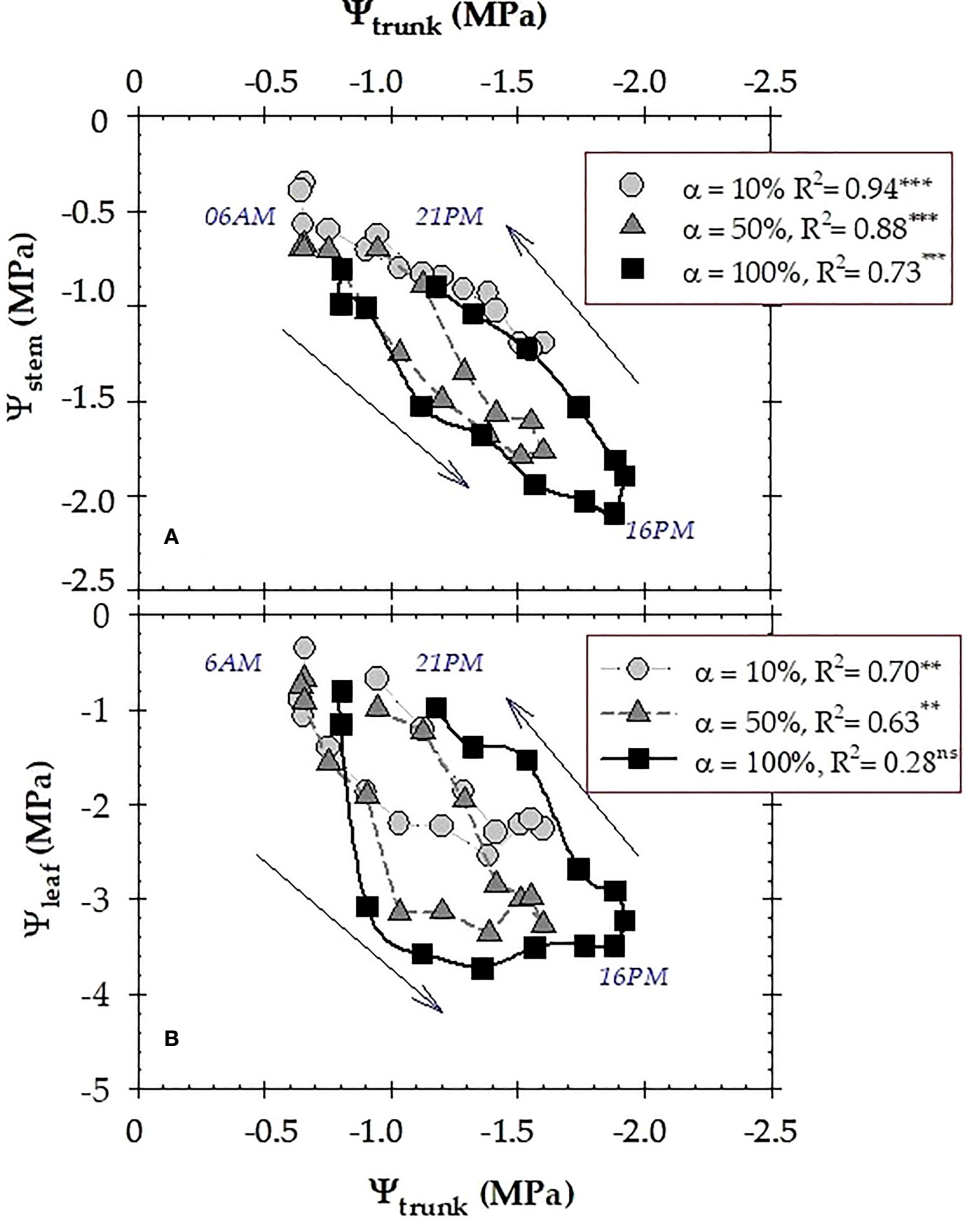
Daily time-course of the relationship between trunk water potential (Ψ_trunk_) and: **(A)** stem water potential (Ψ_stem_) and **(B)** leaf water potential (Ψ_leaf_) at each irrigation criterion: 10% (well-irrigated), 50% (moderate water deficit) and 100% (severe water deficit, non-irrigated), respectively. The data point is the mean of four leaves. R^2^ is the coefficient of determination of the linear regression. **: *p* ≤ 0.01; ***: *p* ≤ 0.001, ns: not significant.

The data in **Figure 8** illustrates the diurnal variations of the relationship of Ψ_trunk_ with Ψ_stem_ (A) and Ψ_leaf_ (B) at the different irrigation periods. Notably, values of Ψ_trunk_ in the early afternoon recovered their morning values at higher Ψ_stem_ values (**Figure 8A**). This fact was more noticeable when considering Ψ_leaf_ values (**Figure 8B**). The significance of the coefficient of determinations was higher during the well irrigated period, and decreased at α=50%, not being significant at α=100% (**Figures 8A, B**).

**Figure 9:**
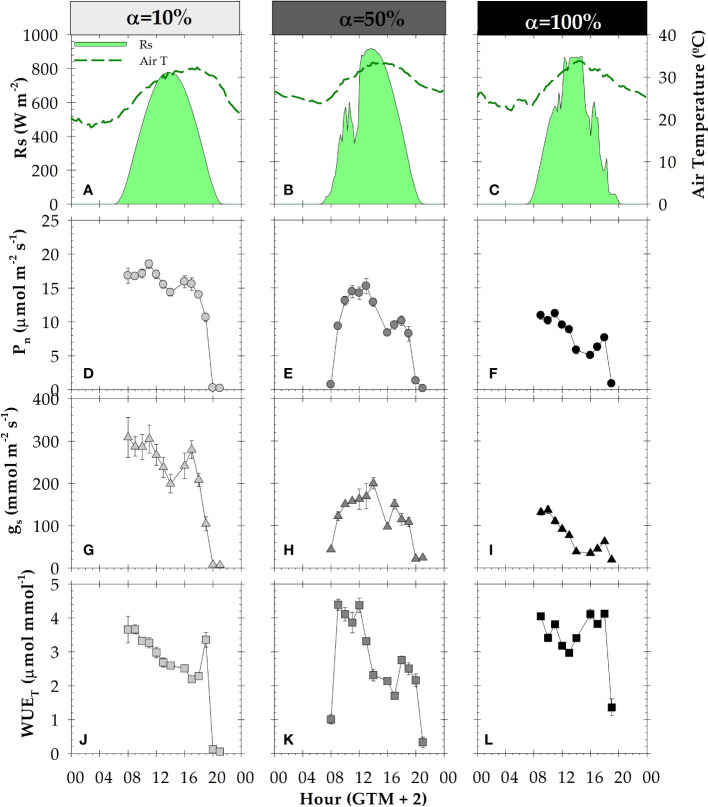
Daily courses of: **(A–C)** solar radiation (R_s_) and air temperature; **(D–F)** net photosynthesis (P_n_); **(G–I)** stomatal conductance (g_s_); **(J–L)** transpiration efficiency (WUE_T_), during different irrigation criteria: α=10% (DOY 174), α=50% (DOY 210) and α=100% (DOY 244). Each point is the mean of four leaves. Vertical bars in data points are ± SE (not shown when smaller than the symbols). GMT: Greenwich mean time.

Regarding daily leaf gas exchange courses, P_n_ and g_s_ increased from sunrise at 08:00 h to 10:30 h GTM+2, which was the period of maximum photosynthetic efficiency in all irrigation conditions studied (**Figure 9**). During midday, leaf gas exchange exhibited a decrease in its values, although it corresponded with the peaks of solar radiation (R_s_) (**Figures 9A–C**). In the afternoon from 16:00 h to 18:00 h GMT+2, leaf gas exchange parameters exhibited a slightly recovery, even under water deficit conditions (α=50 and 100%). From that moment on, the course of leaf gas exchange parameters tended to decrease, until the night hours when minimum values were recorded.

The diurnal patterns of leaf gas exchange followed the established MAD values (**Figures 1B**, **9**). In this sense, at α=10%, the values corresponded to well-irrigated conditions, with maximum values of 17.12 ± 0.65 µmol m^-2^ s^-1^, 308 ± 32.9 µmol m^-2^ s^-1^ and 5.54 ± 0.35 mmol mmol^-1^, for P_n_, g_s_ and WUE_T_, respectively. As expected, the lowest values were obtained under severe water deficit situation (α=100%), with maximum daily values of P_n_ = 10.94 ± 0.60 µmol m^-2^ s^-1^, g_s_ = 137.1 ± 8.60 µmol m^-2^ s^-1^ and WUE_T_ = 4.04 ± 0.08 mmol mmol^-1^.

### 3.4 Osmotic water potentials

**Figure 10:**
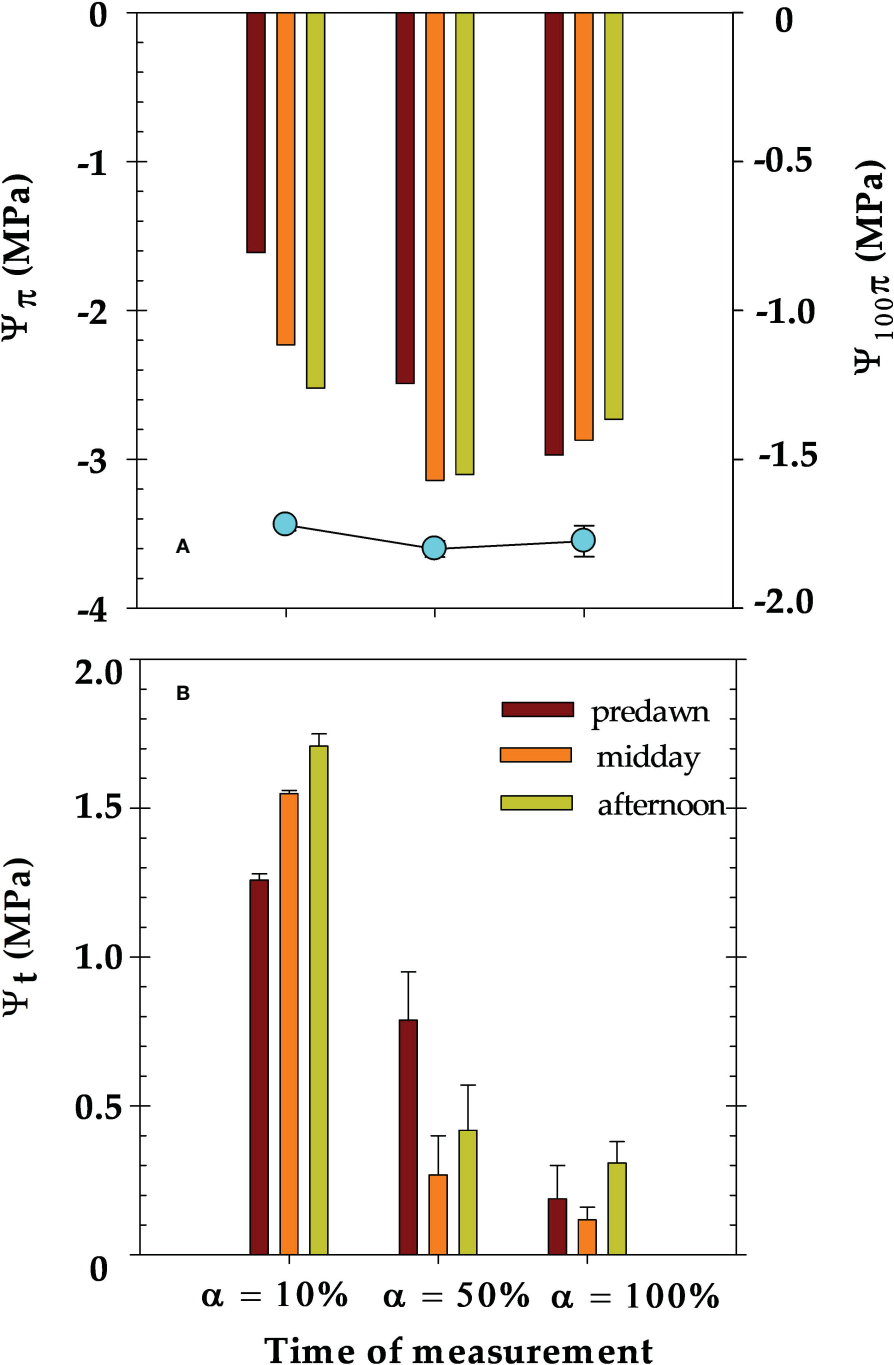
Values of: **(A)** actual osmotic water potential (Ψ_π_), and osmotic water potential at full turgor (Ψ_π100_), and **(B)** and leaf turgor potential (Ψ_t_) at different times of the day (predawn, midday and afternoon) during the different irrigation criteria: α=10% (DOY 174), α=50% (DOY 210) and α=100% (DOY 244). The measurements were made on the same leaves used for leaf water potential. Each bar is the mean of four leaves ± ES.

**Figure 10** shows the values of osmotic water potential (Ψ_π_) determined at different times (predawn, midday and afternoon) during the diurnal courses of the different irrigation criteria. At α=10% (well-irrigated), Ψ_π_ significantly increased from -1.61 ± 0.01 MPa at predawn to -2.97 ± 0.01 MPa in the afternoon. Under water deficit conditions, the minimum Ψ_π_ was found at midday, with values of -3.14 ± 0.05 MPa (at α=50%) and -3.10 ± 0.10 MPa (at α=100%) (**Figure 10A**). In contrast, the osmotic potential at full turgor (Ψ_π100_) measured at predawn was similar throughout the experimental period with a mean value of -1.76 ± 0.03 MPa (**Figure 10A**).

The leaf turgor potential (Ψ_t_) decreased close to zero as soil water deficit increased (**Figure 10B**). In this sense, at α= 100% lower Ψ_t_ values were computed at midday coinciding with the lower leaf water potential (Ψ_leaf_) and higher evaporative demand values (**Figure 6**).

### 3.5 Sensitivity analysis

Comparative analysis of the sensitivity of the indicators of plant water status revealed that plant water potentials showed a higher sensitivity than those obtained for leaf gas exchange (**Table 1**). From the plant water potentials: Ψ_trunk_, Ψ_stem_ and Ψ_leaf,_ it was clear that Ψ_trunk_ was clearly the plant-based water status indicator with the highest SI and sensitivity values by the two methods assessed (S and S*), followed by Ψ_stem_ and to a lesser extent by Ψ_leaf_ and leaf gas exchange parameters (**Table 1**). In particular, CV was slightly lower for Ψ_stem_ (2.15) than for Ψ_trunk_ (2.47). The S was similar between Ψ_trunk_ and Ψ_stem,_ even though S* indicated a higher sensitivity for Ψ_trunk._

**Table 1:** Sensitivity analysis (SI: Signal intensity; CV: coefficient of variation; S: sensitivity (by Goldhamer and Fereres, 2001); and S*: corrected sensitivity (by De la Rosa et al., 2014) for the indicators of plant water status during the experimental period.

Plant water status indicator	SI	CV	S	S*
**Ψ_stem_**	2.13	2.15	0.99	1.67
**Ψ_leaf_**	1.37	5.01	0.27	1.17
**Ψ_trunk_**	2.54	2.47	1.03	2.13
**P_n_**	0.54	2.83	0.19	0.18
**g_s_**	0.39	1.84	0.21	-0.15
**WUE_T_**	0.8	3.08	0.26	0.26

Ψ_stem:_ midday stem water potential (MPa); Ψ_leaf:_ midday leaf water potential (MPa); Ψ_trunk:_ midday trunk water potential (MPa); P_n_: net photosynthesis (µmol m^-2^ s^-1^); g_s_: stomatal conductance (mmol m^-2^ s^-1^); WUE_T_: transpiration efficiency (µmol mmol^-1^).

## 4 Discussion

Continuous recording of trunk water potential (Ψ_trunk_) obtained *in situ* with MTs has been a suitable measure of plant water status of drip-irrigated nectarine trees. Measurements of Ψ_trunk_ have validated the established MAD-based irrigation protocols (**Figure 1B**), becoming useful alternative to discrete measurements of leaf or stem water potentials with traditional pressure chamber (**Figures 2B**, **6**). Moreover, Ψ_trunk_ has the advantage of being measured continuously and in real-time, which could lead to automation, whereas Ψ_leaf_ or Ψ_stem_ are destructive, labour-demanding and time-point measurements (Lakso et al., 2022). Nowadays, the information related to the use of Ψ_trunk_ for irrigation management purposes is scarce, and the few available studies deal with irrigation scheduling based on farmer experience (Pagay, 2022) or ETc requirements (Blanco and Kalcsits, 2021).

In our experiment, automated irrigation, based on MAD threshold values fed by real-time θ_v_ measurements with capacitance probes, has been successfully implemented for drip-irrigated nectarine trees grown in a semi-arid Mediterranean environment. As confirmed in previous studies, significantly higher water, energy and labour savings were achieved using this MAD-based irrigation protocol compared to conventional irrigation scheduling based on calculated crop evapotranspiration (ETc), not only without penalising yield but also improving nectarine fruit quality (Conesa et al., 2019; Conesa et al., 2021; Vera et al., 2019; Vera et al., 2021). In this field experiment, a quite different postulate was applied, in which MAD were managed to reach different soil water deficit conditions, and thus θ_v_ in the active root zone (0-0.5 m) were remained close to FC values during the first period (α=10%), decreased to 21.5% during α=50% period, and barely reached 17% at the end of the withholding irrigation period (α=100%). These θ_v_ values were indicative of well-irrigated, mild and severe soil water deficit conditions, respectively (**Figure 1B**). Since θ_v_ sets the upper/lower interval of the available soil water, θ_v_ variations were due not only in response to irrigation or rainfall events, but also to root water uptake dynamics and, to a lesser extent, diurnal environmental changes. In fact, θ_v_ dynamics had been closely related to evapotranspiration demand, confirming the sensitivity of capacitance sensors to the nearby environment of soil and plant roots (Mira-García et al., 2021).

Water potentials in the soil-plant-atmosphere continuum (SPAC) provide a physical basis for a comparable quantification of water status. During the summer in the northern hemisphere, the agrometeorological measurements were typical of Mediterranean semi-arid climates (Lionello et al., 2023). Of these, air water potential (Ψ_a_) was calculated as an environmental indicator (**Figure 2A**), behaving similarly to VPD (**Figure 1A**), showing a higher day-to-day variability. However, Ψ_a_ gives an indication of the water potential allowing water flow along the soil-plant path.

Soil water status, estimated by soil matric potential (Ψ_m_), also correlated with the irrigation protocol applied (**Figure 1B**). However, under non-irrigated conditions (α=100%), the soil sensors reached their maximum allowed reading (-200 kPa), which mirrored a significant limitation of these soil water sensors under severe water stress conditions (**Figure 2C**). Thompson et al. (2006) reported the best performance of these granular matrix sensors when used in wet soil (-10 to -50 kPa). Also, the pattern of Ψ_m_ at both soil depths (0.3 and 0.6 m) remained almost constant during the daily courses studied (**Figures 6G–I**), highlighting the drawback of these soil water sensors in identifying diurnal changes because of root water uptake.

Plant water potentials understandably reflected the MAD-based irrigation criteria, evaporative demand and radiation changes that occurred throughout the day (**Figures 2B**, **6D–F**). The values of Ψ_leaf_ measured at predawn during the diurnal courses, which decreased as stress accumulated (from -0.35 to -0.8 MPa) (**Figures 6D–F**), agreed with those obtained in deficit irrigated peach trees by Girona et al. (1993). This valued plant-based measurement, taken at night when there is little or no transpiration, gives an indication of the integrated water status of the soil around the roots (Schmidt and Gaudin et al., 2017), based on the idea that when the plant does not transpire, there is a balance between soil and plant water status. However, there can be erroneous values if there are large variations in soil water levels within the profile (Améglio et al., 1999).

The values of Ψ_leaf_ showed the highest variability of the plant water potentials studied (**Figures 2B**, **6D–F**). This is because it is determined on non-cover sunlit leaves, highly dependent upon leaf conductance values and evaporative demand conditions existing at the time of the measurements (García-Tejara et al., 2021). In this sense, Ruiz-Sánchez et al. (2000) found a strong relationship between leaf insertion angle (LIA) and Ψ_leaf_ in apricot trees, so that the variability in Ψ_leaf_ caused by changes in leaf orientation allows a lower incidence of solar radiation, and a reduction in water loss and leaf heating (Sánchez-Blanco et al., 1994), which makes sunny leaves sensitive to the time of sun exposure. Consequently, Ψ_stem_, measured on covered leaves, is considered the standard measure to determine tree water status in fruit trees (Shackel et al., 1997). Since leaf transpiration is prevented, the Ψ_stem_ roughly represents soil water status, and behaved more stable than Ψ_leaf_ (**Figures 2B**, **6D–F**).

In the present study, both Ψ_trunk_ and Ψ_stem_ were strongly correlated (R^2^ = 0.86, *p*<0.001), as they provided similar data of plant water path (**Figure 4A**). Blanco and Kalcsits (2021) found similar correlations with a coefficient of determination up to 0.8 in pear trees. However, the relationship between Ψ_trunk_ *vs.* Ψ_leaf_ was not significant, highlighting the higher Ψ_leaf_ variability and the weakness of this indicator of plant water status (**Figure 4B**).

Seasonal values of Ψ_stem_ and Ψ_trunk_ averaged -0.83 and -0.73 MPa, respectively, during the period of α=10% (**Figure 2B**), coinciding with the postharvest period in nectarine trees. These values corresponded to non-limiting soil water conditions (Naor et al., 2005; Abrisqueta et al., 2015; De la Rosa et al., 2016; Conesa et al., 2019; Vera et al., 2019, **Conesa et al., 2021**). As expected, the minimum values of Ψ_stem_ (-2.04 MPa) and Ψ_trunk_ (-1.74 MPa) were observed at the end of the non-irrigation period (α=100%) (**Figure 2B**). Blanco and Kalcsits (2021) reported that MTs can accurately assess plant water status within the range of -0.2 to -2.1 MPa of Ψ_trunk_ values in pear trees. Our findings showed a mean gradient of 0.3 MPa between Ψ_stem_ and Ψ_trunk_ (**Figures 2B**, **6D–F**, **7**), with slight differences during the experiment. Also, the gradient between Ψ_leaf_ and Ψ_trunk_ was higher than that between Ψ_stem_ and Ψ_trunk_ (mean values of ≈ 1.8 MPa), indicative of the high hydraulic resistance between trunk and leaves (Pagay, 2022).

It is noteworthy that when seasonal data of plant and soil water potentials were correlated, the most significant relationship was detected between Ψ_m_ *vs.* Ψ_trunk_ (R^2^ = 0.79, *p*<0.01) followed by Ψ_m_ *vs.* Ψ_stem_ (R^2^ = 0.62, *p*<0.05) and it was not significant for Ψ_m_ *vs.* Ψ_leaf_ (R^2^ = 0.26, *p*>0.05) (**Figure 3**). Thus, it reveals that Ψ_trunk_ is arguably the most stable indicator of the plant water status, integrating canopy leaves into a stable tissue relatively unaffected by external factors (Lakso et al., 2022; Pagay, 2022).

Leaf gas exchange was also sensitive to MAD-based irrigation criteria (**Figure 5**). As expected, P_n_ and g_s_ decreased during the experiment as water deficit accumulated, suggesting a limitation in photosynthetic capacity under water stress condition (Wong et al., 1979). Meanwhile, transpiration efficiency (WUE_T_) tended to increase (**Figure 5C**). Stomatal closure (**Figure 5B**) reduced the amount of H_2_O lost per CO_2_ assimilated, although, the response of this plant indicator to water stress was decreased by the effect of climatic demand (**Figures 5C**, **1A**). It is also important to note that, despite irrigation recovery, mean values of P_n_ and g_s_ at this period were lower than those obtained under early summer well irrigated conditions (α=10%). The absence of a full recovery of leaf gas exchange values was motivated by the initiation of leaf senescence typical of deciduous fruit trees (Andersen and Brodbeck, 1988). Furthermore, Conesa et al. (2022) explained the fact that leaf gas exchange levels of water stressed nectarine trees during late postharvest did not recover previous values, after irrigation was restored, by a decrease in the aspartate amino acid in leaves that affected chloroplasts formation.

The hysteresis phenomenon found in the relationships between the two plant water potentials: Ψ_trunk_ *vs.* Ψ_stem_ (**Figure 8A**), which was more noticeable for Ψ_trunk_ *vs.* Ψ_leaf_ (**Figure 8B**), was higher for the highest imposed soil water deficit (α=100%). This hysteretic behaviour revealed that the water status of the trunk assumes a dominant role in controlling canopy water status as water stress accumulates, which is related to plant hydraulic conductivity during the daily course (Assouline, 2021). In addition, stomata reopened in the afternoon, as indicated by the recovery values of the diurnal pattern of leaf gas exchange (**Figures 9G–I**).

No osmotic adjustment was observed in leaves of nectarine trees in response to the applied soil water deficit (**Figure 10A**). In this regard, Mellisho et al. (2011) in peach trees, and Torrecillas et al. (1999) in apricot trees reported the need to reach Ψ_leaf_ and Ψ_stem_ below -2.6 and -2.0 MPa, respectively, to activate this tolerance mechanism. Furthermore, it was observed that leaf turgor (Ψ_t_) was maintained, even at α=100% (**Figure 10A**). In this sense, other drought tolerance characteristics could have taken place, such as high relative apoplastic water content, which would contribute to water retention at low leaf water potentials (Rodríguez et al., 2012).

It is important to note that Ψ_trunk_ values showed the highest signal intensity and sensitivity values for the plant-based water status indicators studied, followed by Ψ_stem_ (**Table 1**). These results emphasise that although Ψ_trunk_ had a higher variability (CV) than Ψ_stem_, it can accurately assess plant water status. Indeed, the S* method (De la Rosa et al., 2014), which decreased the influence of CV in the analysis, showed an increased sensitivity of Ψ_trunk_. In the same cultivar, Ψ_stem_ and canopy to air temperature difference values recorded the highest signal intensity and the Normalised Difference Vegetation Index the highest sensitivity for detecting moderate water deficit situations by mid-July (Conesa et al., 2019).

## 5 Conclusions

Continuous measurements of trunk water potential (Ψ_trunk_) using microtensiometers, embedded in the tree trunk, agreed with the automated soil MAD-based irrigation protocols applied to a nectarine orchard. Changes in Ψ_trunk_ explained 79% of the soil matric potential. In fact, Ψ_trunk_ was strongly related to discrete determinations of Ψ_stem_ measured with a pressure chamber. A mean gradient of 0.3 MPa was observed between Ψ_trunk_ *vs.*Ψ_stem_, and 1.8 MPa between Ψ_trunk_ *vs.*Ψ_leaf_. The greatest variability was found in Ψ_leaf_, due to its dependence on stomatal aperture and evaporative demand conditions. Regarding environmental variables, Ψ_air_ showed a high day-to-day variability and a similar dynamic to VPD. Therefore, Ψ_air_ could be used in water relations studies in the same terms of water potential as in soil and plant.

Considering that real-time Ψ_trunk_ data allows for automation, further research is needed to determine Ψ_trunk_ threshold values for a successful irrigation decision support system. In addition, the stability, and the long-term performance of trunk microtensiometers needs to be tested.

The promising results found in this work point to the potential use of trunk microtensiometers as novel biosensors to accurately real-time monitor plant water status, and eventually served for precise irrigation scheduling.

## Data availability statement

The raw data supporting the conclusions of this article will be made available by the authors, without undue reservation.

## Author contributions

Study conception and design were performed by MC, JV and MR-S. Formal analysis and data curation by WC and MC. Software and validation by JV and WC. Project administration and funding acquisition by MR-S. The first draft of the manuscript was written by MC. All authors contributed to the article and approved the submitted version.
